# Predicting the risk of neurocognitive decline after brain irradiation in adult patients with a primary brain tumor

**DOI:** 10.1093/neuonc/noae035

**Published:** 2024-04-10

**Authors:** Fariba Tohidinezhad, Catharina M L Zegers, Femke Vaassen, Jeanette Dijkstra, Monique Anten, Wouter Van Elmpt, Dirk De Ruysscher, Andre Dekker, Daniëlle B P Eekers, Alberto Traverso

**Affiliations:** Department of Radiation Oncology (Maastro Clinic), School for Oncology and Reproduction (GROW), Maastricht University Medical Center, Maastricht, The Netherlands; Department of Radiation Oncology (Maastro Clinic), School for Oncology and Reproduction (GROW), Maastricht University Medical Center, Maastricht, The Netherlands; Department of Radiation Oncology (Maastro Clinic), School for Oncology and Reproduction (GROW), Maastricht University Medical Center, Maastricht, The Netherlands; Department of Medical Psychology, School for Mental Health and Neurosciences (MHeNS), Maastricht University Medical Center, Maastricht, The Netherlands; Department of Neurology, School for Mental Health and Neuroscience (MHeNS), Maastricht University Medical Center, Maastricht, The Netherlands; Department of Radiation Oncology (Maastro Clinic), School for Oncology and Reproduction (GROW), Maastricht University Medical Center, Maastricht, The Netherlands; Department of Radiation Oncology (Maastro Clinic), School for Oncology and Reproduction (GROW), Maastricht University Medical Center, Maastricht, The Netherlands; Department of Radiation Oncology (Maastro Clinic), School for Oncology and Reproduction (GROW), Maastricht University Medical Center, Maastricht, The Netherlands; Department of Radiation Oncology (Maastro Clinic), School for Oncology and Reproduction (GROW), Maastricht University Medical Center, Maastricht, The Netherlands; Department of Radiation Oncology (Maastro Clinic), School for Oncology and Reproduction (GROW), Maastricht University Medical Center, Maastricht, The Netherlands; School of Medicine, Libera Università Vita-Salute San Raffaele, Milan, Italy

**Keywords:** brain neoplasms, cognitive dysfunction, cranial irradiation, machine learning, neurotoxicity

## Abstract

**Background:**

Deterioration of neurocognitive function in adult patients with a primary brain tumor is the most concerning side effect of radiotherapy. This study aimed to develop and evaluate normal-tissue complication probability (NTCP) models using clinical and dose–volume measures for 6-month, 1-year, and 2-year Neurocognitive Decline (ND) postradiotherapy.

**Methods:**

A total of 219 patients with a primary brain tumor treated with radical photon and/or proton radiotherapy (RT) between 2019 and 2022 were included. Controlled oral word association test, Hopkins verbal learning test-revised, and trail making test were used to objectively measure ND. A comprehensive set of potential clinical and dose–volume measures on several brain structures were considered for statistical modeling. Clinical, dose–volume and combined models were constructed and internally tested in terms of discrimination (area under the curve, AUC), calibration (mean absolute error, MAE), and net benefit.

**Results:**

Fifty percent, 44.5%, and 42.7% of the patients developed ND at 6-month, 1-year, and 2-year time points, respectively. The following predictors were included in the combined model for 6-month ND: age at radiotherapy > 56 years (OR = 5.71), overweight (OR = 0.49), obesity (OR = 0.35), chemotherapy (OR = 2.23), brain *V*_20 Gy_ ≥ 20% (OR = 3.53), brainstem volume ≥ 26 cc (OR = 0.39), and hypothalamus volume ≥ 0.5 cc (OR = 0.4). Decision curve analysis showed that the combined models had the highest net benefits at 6-month (AUC = 0.79, MAE = 0.021), 1-year (AUC = 0.72, MAE = 0.027), and 2-year (AUC = 0.69, MAE = 0.038) time points.

**Conclusions:**

The proposed NTCP models use easy-to-obtain predictors to identify patients at high risk of ND after brain RT. These models can potentially provide a base for RT-related decisions and post-therapy neurocognitive rehabilitation interventions.

## Graphical Abstract

**Graphical Abstract:**
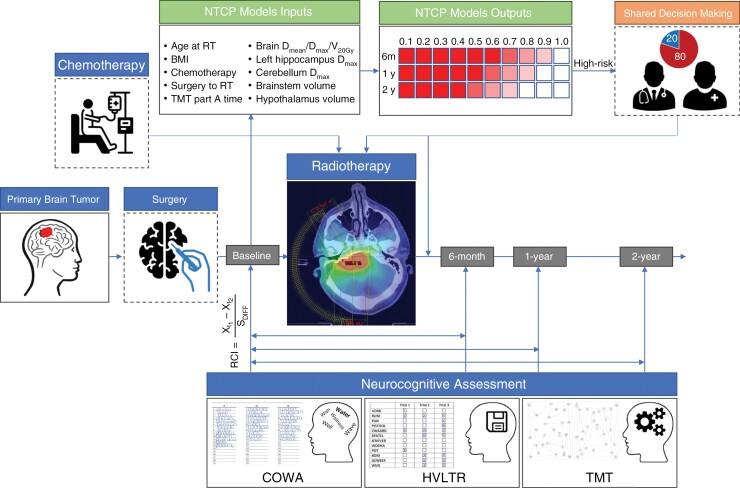

**Key Points**

- Hippocampus and cerebellum sparing during RT planning can reduce the risk of ND.
- BMI, brainstem, and hypothalamus volumes were negatively associated with ND.
- Proposed NTCP models can be used as simple tools to facilitate the shared decision-making process.

**Importance of the Study**

Current national and international guidelines use thresholds to spare critical brain and head structures during radiotherapy planning. Considering neurocognitive decline as the most prevalent side effect of radiotherapy, we have developed comprehensive risk stratification models which take into account all protective/risk factors in the patient’s profile (sociodemographic, tumor specifications, previous cancer treatments, medication use, baseline neurocognitive function, and radiotherapy dose–volume measures), and provide accurate individualized risk estimations. Risk assessment at different time points (6-month, 1-year, and 2-year) will help clinicians to identify the patients who are at high risk of persistent decline and therefore need close monitoring during postradiotherapy follow-up visits and may potentially benefit from neurocognitive rehabilitation therapy. Moreover, our findings suggest that left hippocampus *D*_max_ and cerebellum *D*_max_ were risk factors for 1-year and 2-year ND, which can be incorporated into optimizing a treatment plan and potentially reduce the risk of neurocognitive decline.

Brain Radiotherapy (RT) remains a mainstay therapeutic modality for benign and malignant brain tumors either as primary or adjuvant treatment combined with surgery, chemotherapy, and/or molecularly targeted agents.^1^ Radiation dose to the brain, however, can cause neuroinflammation and demyelination, inhibit synaptic plasticity and neurogenesis^2^ and disrupt functional brain networks which play an important role in neurocognition.^3^ It has been shown that Neurocognitive Decline (ND) after brain RT affects 50–90% of the patients, appearing as early as 3–4 weeks or with longer latency periods.^4^ The early forms of ND can persist and synergize over time to cause irreversible deficits which may limit the patient’s ability to perform daily activities.^5^

In recent years, ND has been prioritized as an integrated clinical endpoint alongside tumor control and survival measures to quantify the quality of life of patients with primary brain tumors.^6^ Depending on the aim of the assessment, several subjective and objective instruments are available to quantify the level of ND. Previous studies show that patients with primary brain tumors may overestimate their neurocognitive capacities due to impaired judging ability or alternatively, they may underestimate their neurocognitive functions due to accompanied disease-related feelings of fatigue, anxiety, or depression.^7^ Therefore, it is recommended to use a comprehensive series of standard objective tests. The Controlled Oral Word Association (COWA) test, Hopkins Verbal Learning Test-Revised (HVLTR), and Trail Making Test (TMT) have shown sensitivity in detecting neurotoxic effects of cancer treatments.^8^ These tests allow for measuring vast amounts of information on different neurocognitive domains (ie language abilities, memory, learning, processing speed, attention, and executive function) quantifying the functions of both brain hemispheres.^9^

Since the underlying mechanism of ND after brain irradiation is poorly understood, its protective and risk factors have not yet been completely elucidated.^10^ Sparse findings are available suggesting that radiation dose to certain brain structures such as the hippocampus^11–13^ or cerebellum^14,15^ increases the risk of ND. Several studies have shown that it may also be a consequence of clinical factors which have a potential impact on further deterioration.^16^ Therefore, validated Normal-Tissue Complication Probability (NTCP) models are needed for model-based decisions on the applied radiotherapy technique, treatment planning, and post-RT patient monitoring in clinical practice. However, to the best of our knowledge, a comprehensive NTCP model for adult patients is still lacking.^17^ Available NTCP models mainly predict the intelligence quotient of pediatric survivors following brain irradiation.^18^

This study aimed to develop and evaluate NTCP models for ND assessed by the COWA, HVLTR, and TMT in adults affected by a primary brain tumor at 6 months, 1 year, and 2 year after first-line or postoperative brain RT administered with standard fractionation. Dose–volume parameters of several brain structures were analyzed to identify their predictive value. Moreover, the potential association of ND and Overall Survival (OS) was examined.

## Materials and Methods

### Study Population

This retrospective study included adult patients with a primary brain tumor treated with radical fractionated photon and/or proton RT between May 2019 and December 2022 at the Department of Radiation Oncology of the Maastricht University Medical Center (Maastro Clinic). The inclusion criteria were low-grade or high-grade primary brain tumors, age ≥ 18 years and Karnofsky Performance Status (KPS) ≥ 70%. Patients with previous cranial irradiation, hypo-fractionated stereotactic irradiation, survival of less than 6 months post-RT or lack of compliance with neurocognitive assessments were excluded. The survival status of the patients was automatically retrieved from the Dutch national personal records database (Basisregistratie Personen, BRP). The patients were censored either due to the study end date or brain re-irradiation for tumor progression. The study was approved by the Internal Review Board of the Maastro Clinic (W210800051).

### Radiation Treatment

The pretreatment (planning) Computed Tomography (CT) images were rigidly registered with fluid-attenuated inversion recovery and T1-weighed magnetic resonance images with a contrast agent. Delineation of Gross Tumor Volume (GTV) and Clinical Target Volume (CTV) was performed by experienced radiation oncologists specialized in neuro-oncological radiotherapy. The Organs-at-risk (OARs) were defined according to the European Particle Therapy Network consensus-based atlas for contouring in neuro-oncology.^19^ The target volumes (GTVs and CTVs) and fractionation schedules were defined according to national and international guidelines, using a GTV to CTV margin of 0–1.5 cm depending on the tumor characteristics.^20^ Photon and proton treatment planning were performed using Eclipse v11 (Varian Medical Systems) and RayStation v10A (RaySearch Laboratories), respectively, considering 1.1 relative biological effectiveness. Prescribed dose ranged from 40 to 60 Gy with 1.8, 2 or 2.67 Gy fraction dose. Patient positioning was verified before every fraction using either kilovoltage images or cone beam CT.

### Potential Clinical Predictors

The following clinical variables were selected as candidates for statistical modeling based on published papers^17^: age, gender, Body Mass Index (BMI), social status (education, partnership and cohabitation), tumor specifications (World Health Organization (WHO) grade, histology, laterality and location), cancer treatments (surgery and chemotherapy), radiotherapy (modality, prescribed dose, fraction dose and duration), comorbidities (diabetes, hypertension, hyperlipidemia, cerebrovascular, psychological, autoimmune, thyroid and ocular disorders), substance abuse, medication profile, performance status, and physical manifestations (seizure, amnesia, dizziness and headache) measured by Common Terminology Criteria for Adverse Events (CTCAE v.4).

### *Dose*–*Volume Parameters*

Total brain structure, hippocampus (left, right and entire structure), hypothalamus (left, right and entire structure), brainstem (interior, surface and entire structure), cerebellum, and pituitary gland were considered for dose–volume extraction. The dose–volume parameters were extracted for the substructures excluding the CTV (substructure-CTV) to consider the volume and dose received by the healthy tissues. The following measures were extracted for each OAR: minimum, mean, and maximum dose to the structure in Gy, dose received by *x*% volume of the structure (*D*_*x*_), structure volume in cc, volume of the structure in % receiving *x*Gy (*V*_*x*_) and dose to 2% and 98% of the CTV in Gy representing near maximum and minimum doses received by the target.

### Endpoint Definition

Baseline neurocognitive function was measured prior to RT. Post-RT assessments were performed during regular clinical follow-up visits at 6 months and thereafter on a yearly basis.^21^ The following 3 tests were used: COWA for lexical verbal fluency,^22^ HVLTR for memory (both immediate and delayed recall)^23^ and TMT (parts A and B) for visual and spatial scanning, sequencing, attention, speed, and executive skills.^24^ This shortened battery was selected in accordance with the collaboration agreement of the Neuro Dutch Proton Therapy Centers (DUPROTON), which was consistent with the recommendations from the International Cognition and Cancer Task Force (ICCTF) and Response Assessment in Neuro-Oncology (RANO) on outcome evaluation of radiation toxicity.^9,25^ The recently published consensus by the European Particle Therapy Network (EPTN) also confirmed the use of COWA, HVLTR and TMT as the core tests for neurocognitive assessment in adult patients with brain tumors receiving RT.^8^ To minimize the burden on patients, the 3 tests were conducted within a 30-min timeframe.

The Reliable Change Index (RCI) was calculated using the following equation to identify the change that is unlikely to occur due to error of measurement^26^:


$$\begin{array}{l} RCI=\frac{Score_{baseline}-Score_{Follow\text{\textasciitilde{}}\text{-}up}}{\sqrt{2{\left(s\sqrt{1-r}\right)}^{2}}} \end{array}$$

where *s* is the SD in the reference group and *r* is the test–retest Cronbach alpha from the literature.^22–24^ Patients with an RCI (COWA) > 1.5 or RCI (HVLTR) > 1.5 or RCI (TMT) < –1.5 were considered as reliable deterioration.

### Statistical Analysis

Univariable analysis was performed to determine the prognostic value of the clinical and dose–volume variables. Multivariable logistic regression with backward selection based on the Akaike information criterion was used to develop the clinical model. To identify the most robust dose–volume signature (more than 1 predictor), multivariable analysis was performed on 1000 bootstrap resamples. The top frequent signature was selected and the coefficients were then fitted using the original data. Significant predictors in the clinical and dose–volume models were used to build the combined models for 6-month, 1-year and 2-year ND. An Events Per Predictor (EPP) ≥ 14 was considered to reduce the risk of overfitting. Dose–volume measures were dichotomized based on the optimal threshold to discriminate the patients with and without ND. The predictive performance of the models was quantified in terms of discrimination (area under the receiver operating characteristic curve (AUC)) and calibration (mean absolute error (MAE) and calibration plot). Positive predictive value (PPV) and negative predictive value (NPV) were calculated based on the Youden index threshold determination. The AUC and calibration measures were corrected for optimism using 1000 bootstrap samples. The decision curve analysis (DCA) was used to calculate the $Net\;Benefit=\frac{TP-FP}{N}\times \frac{P_{t}}{1-P_{t}}$ (where TP is true positive, FP is false positive, *P*_*t*_ is the threshold of predicted probability and *N* is the number of patients) of the prediction models and compare their clinical utility in decision-making process with the 2 benchmarking strategies of “treat none and “treat all.”^27^

Nomograms of the combined models were constructed based on the multivariable equations. The Kaplan–Meier with log-rank test was used to assess the difference in OS between the patients with and without 6-month ND. All analyses were performed in R v.4.3.1 (R Foundation for Statistical Computing).

## Results

A total of 219 patients (age at RT initiation: 54.4 ± 14.9 years, 47% male) were included. Histological types were meningioma (*n* = 56, 26%), glioblastoma (*n* = 49, 22%), astrocytoma (*n* = 43, 20%), oligodendroglioma (*n* = 35, 16%) and other (*n* = 36, 16%). WHO tumor grades were 1 in 51 (23%), 2 in 78 (36%), 3 in 30 (14%), and 4 in 49 (22%) patients. The tumor was located in the left or right hemispheres in 88 (40%) and 111 (51%) patients, respectively. The predominant tumor location was in the frontal (*n* = 82, 37%), temporal (*n* = 45, 21%), and parietal (*n* = 29, 13%) lobes. Tumor resection was performed in 165 (75%) patients prior to RT. Sequential or concurrent–sequential chemotherapy was performed in 74 (34%) and 51 (23%) patients, respectively. Chemotherapy agents were temozolomide in 91 (42%) and procarbazine, lomustine, and vincristine in 34 (16%) patients. Photon, proton or combined RT was performed for 118 (54%), 24 (11%) and 77 (35%) patients, respectively. Further statistics on the study sample are presented in Table 1.

**Table 1.** Baseline Sociodemographic, Tumor, Treatment, Comorbidities and Medication Use of the Study Sample

Variable	*N* = 219
Age at radiotherapy	
≤56	118 (54%)
>56	101 (46%)
Male gender	102 (47%)
Body mass index	
Normal	65 (30%)
Overweight	101 (46%)
Obese	53 (24%)
Education level	
Low	61 (28%)
Middle	74 (34%)
High	84 (38%)
Living alone	65 (30%)
WHO grade	
I	51 (23%)
II	78 (36%)
III	30 (14%)
IV	49 (22%)
No grade	11 (5%)
Laterality	
Left	88 (40%)
Right	111 (51%)
Midline	20 (9.1%)
Location	
Frontal	82 (37%)
Temporal	45 (21%)
Parietal	29 (13%)
Base of skull	22 (10%)
Other	41 (19%)
Histology	
Meningioma	56 (26%)
Glioblastoma	49 (22%)
Astrocytoma	43 (20%)
Oligodendroglioma	35 (16%)
Other	36 (16%)
Surgery	
None	30 (14%)
Biopsy	24 (11%)
Resection	165 (75%)
Chemotherapy	
None	94 (43%)
Yes	125 (57%)
Modality of radiotherapy	
Photon	118 (54%)
Proton	24 (11%)
Photon and proton	77 (35%)
Prescribed dose (Gy)	
40.05–46.8	22 (10%)
50.4–52.2	88 (40%)
54–59.4	71 (32%)
60	38 (17%)
Fraction dose (Gy)	
1.8	170 (78%)
2	38 (17%)
2.67	11 (5%)
Karnofsky performance score (%)	
90–100	132 (60%)
70–80	87 (40%)
Diabetes	
IDDM	11 (5%)
NIDDM	11 (5%)
Hypertension	74 (34%)
Hyperlipidemia	47 (21%)
Cerebrovascular diseases	29 (13%)
Psychological disorders	33 (15%)
Smoking	
Current	23 (11%)
Former	76 (35%)
Antithrombotic	20 (9.1%)
Antiepileptic	81 (37%)
Steroid	49 (22%)

**Abbreviations:** IDDM, insulin-dependent diabetes mellitus; NIDDM, noninsulin-dependent diabetes mellitus; WHO, World Health Organization.

The overall 1-year and 2-year survival rates were 91% and 82% with the median follow-up of 26 (19–39) months. Patients with WHO grade IV had 1-year and 2-year survival rates of 62% and 38%, whereas patients with grade I, II, and III presented a 100% 1-year survival rate and 98%, 96%, and 88% survival rates at 2-year time point (Supplementary Figure 1). The majority of the observed deaths were attributed to cancer-related causes and 4 patients died due to noncancer events. Thirteen patients were censored due to re-irradiation for tumor progression.

**Figure 1.**
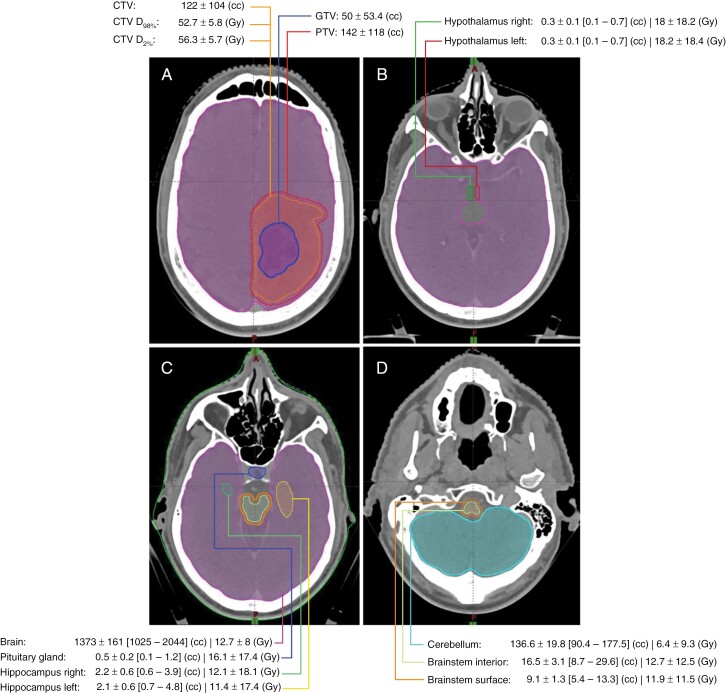
Descriptive statistics on the dose-volume parameters.

The mean dose to the total brain was 12.7 ± 8 Gy. Hypothalamus (left: 18.2 ± 18.4 Gy, right: 18 ± 18.2 Gy), pituitary gland (16.1 ± 17.4 Gy) and interior brainstem (12.7 ± 12.5 Gy) received the highest RT doses. The mean GTV was 50 ± 53.4 cc and the mean CTV D2% and D98% were 56.3 and 52.7 Gy, respectively. The mean dose and volume measures are shown in Figure 1.

ND occurred in 103/206 (50%) patients at 6-month, 72/162 (44.5%) patients at 1-year and 44/103 (42.7%) patients at 2-year time points. The decline in verbal fluency (COWA) occurred in 23 (11.2%), 9 (5.6%), and 8 (7.8%) patients, memory functions (HVLTR) was declined in 56 (27.2%), 43 (26.5%) and 22 (21.4%) patients and TMT showed a decline in 67 (32.5 %), 43 (26.5%), and 26 (25.2%) patients at 3 time points, respectively. A total of 13, 37, and 22 eligible patients did not perform 6-month, 1-year, and 2-year assessments, respectively. The reasons, ranked by frequency, were: no appointment attendance, illness, patient refusal or other reasons.

**Figure 2.**
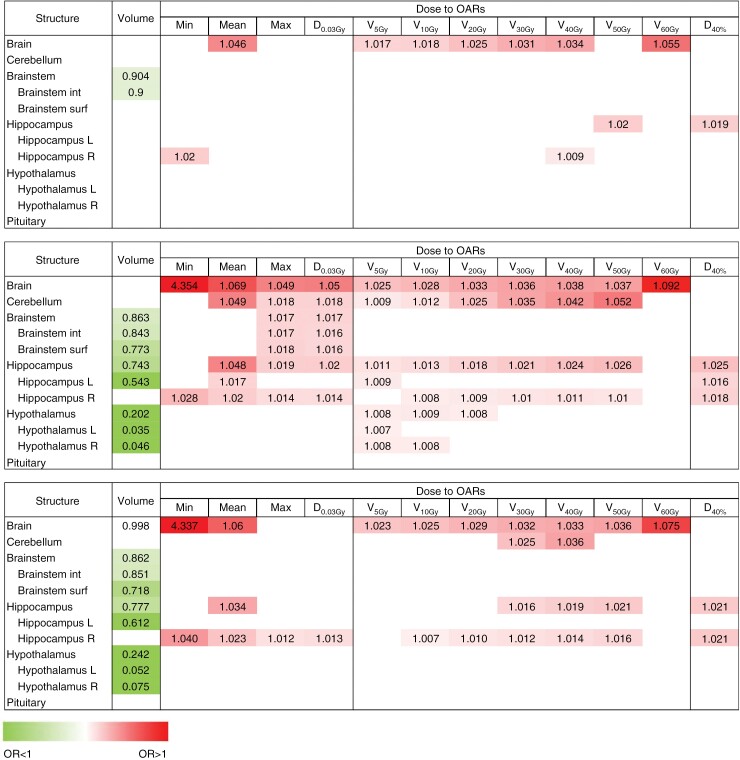
Univariable analysis of the dose-volume measures.

Univariable analysis of clinical variables is presented in Supplementary Table 2. Dose–volume univariable associations showed that brain *V*_5 Gy_–*V*_60 Gy_ and dose to the cerebellum and right hippocampus were positively associated with 6-month ND. The volume of the brain, brainstem, hippocampus, and hypothalamus showed inverse associations with 6-month ND. Moreover, dose to the brain and hippocampus were found to increase the risk of a 6-month decline in HVLTR scores. Detailed univariable associations on dose–volume measures are shown in Figure 2.

The following predictors were included in the clinical NTCP model for 6-month ND: age at radiotherapy > 56 years (OR = 6.43, 95% CI: 3.11–8.32), male gender (OR = 0.42, 95% CI: 0.22–0.81), obesity (OR = 0.45, 95% CI: 0.18–1.09), a high education level (OR = 0.48, 95% CI: 0.21–1.09), chemotherapy (OR = 5.13, 95% CI: 2.41–7.93). Significant dose–volume measures for predicting 6-month ND were: brain *V*_20 Gy_ (≥20% vs <20%) OR = 3.57 (95% CI: 1.92–6.62), brainstem volume (≥26 cc vs <26 cc) OR = 0.36 (95% CI: 0.2–0.67) and hypothalamus volume (≥0.5 cc vs <0.5 cc) OR = 0.38 (95% CI: 0.2–0.7). After integrating the clinical and dose–volume measures, gender and education levels were dropped out from the combined model (Table 2).

**Table 2.** Clinical, Dose–Volume and Combined Models for Predicting the Risk of 6-Month Neurocognitive Decline in Patients with a Primary Brain Tumor Treated with Radiotherapy

Clinical Model	OR (95% CI)	*P*	Combined Model	OR (95% CI)	*P*
(Intercept)	0.55 (0.2–1.53)	.3	(Intercept)	0.69 (0.26–1.85)	.5
Age at radiotherapy (>56 vs ≤56)	6.43 (3.11–8.32)	<.001	Age at radiotherapy (>56 vs ≤56)	5.71 (2.69–7.13)	<.001
Gender (male vs female)	0.42 (0.22–0.81)	0.009	Body mass index		
Body mass index			Normal	[Reference]	
Normal	[Reference]		Overweight	0.49 (0.22–1.08)	.075
Overweight	0.53 (0.25–1.14)	.1	Obese	0.35 (0.14–0.88)	.026
Obese	0.45 (0.18–1.09)	.076	Chemotherapy		
Education level			No	[Reference]	
Low	[Reference]		Yes	2.23 (0.92–5.43)	.077
Middle	0.86 (0.38–1.96)	.7	Brain *V*_20 Gy_ (≥20% vs < 20%)	3.53 (1.53–6.15)	.003
High	0.48 (0.21–1.09)	0.08	Brainstem volume (≥26cc vs <26cc)	0.39 (0.2–0.75)	0.005
Chemotherapy			Hypothalamus volume (≥0.5cc vs <0.5cc)	0.4 (0.2–0.79)	0.008
No	[Reference]				
Yes	5.13 (2.41–7.93)	<.001			
**Dose**–**volume model**	**OR (95% CI)**	***P***			
(Intercept)	1.41 (0.81–2.46)	.2			
Brain *V*_20 Gy_ (≥20% vs < 20%)	3.57 (1.92–6.62)	<.001			
Brainstem volume (≥26 cc vs <26 cc)	0.36 (0.2–0.67)	.001			
Hypothalamus volume (≥0.5 cc vs <0.5 cc)	0.38 (0.2–0.7)	.002			

Patients with a tumor in the temporal lobe, brain *D*_mean_ ≥ 10 Gy and left hippocampus *D*_max_ ≥ 7 Gy were shown to be at higher risk of 1-year ND. The longer duration between surgery and radiotherapy, high education level and brainstem interior volume ≥ 16 cc were found to be protective factors for 1-year ND. Details on the clinical, dose–volume and combined prediction models at 1-year are presented in Supplementary Table 3.

At the 2-year timepoint, patients with middle education level (versus low) had a significantly lower risk of developing ND. Brain *D*_max_ ≥ 54 Gy (versus <54 Gy), cerebellum *D*_max_ ≥ 27 Gy (versus <27 Gy) and TMT part A time > 32 s (≤32 s) were found to increase the risk of 2-year ND (Supplementary Table 5).

Apparent and optimism-correct AUCs of the combined model for 6-month ND were 0.81 and 0.79 (95% CI: 0.76–0.87), respectively. The discrimination power of the combined models for 1-year (apparent: 0.75, optimism-corrected: 0.72) and 2-year (apparent: 0.72, optimism-corrected: 0.69) timepoints were slightly lower compared to the 6-month timepoint. The PPV and NPV of the combined models were 75% and 73% at 6-month, 75% and 71% at 1-year, and 57% and 76% at 2-year timepoints.

The combined model at 6 months showed good individual-based predictions with an MAE of 0.021 on the bootstrap samples. Accordingly, the calibration curve conformed to the ideal line across the entire range of predicted probabilities. At 1-year and 2-year time points the MAEs were slightly higher and the predicted probabilities were bounded to 0.1–0.8.

**Figure 3.**
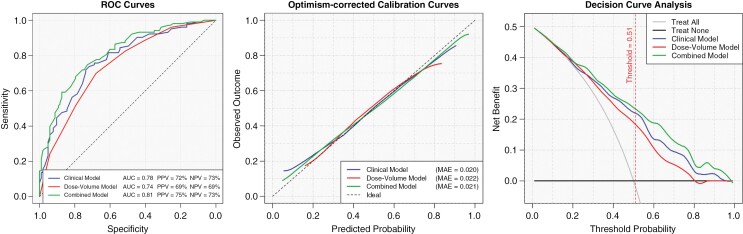
Performance measures of the prediction models.

The DCA revealed the higher net benefits of the combined models across the majority of the threshold probabilities compared with the clinical and dose–volume models at 3 time points. The optimal thresholds for binary risk stratification are shown on decision curves. Performance measures of the models for 6-month, 1-year, and 2-year ND are shown in Figure 3, Supplementary Figures 4 and 6, respectively.

**Figure 4.**
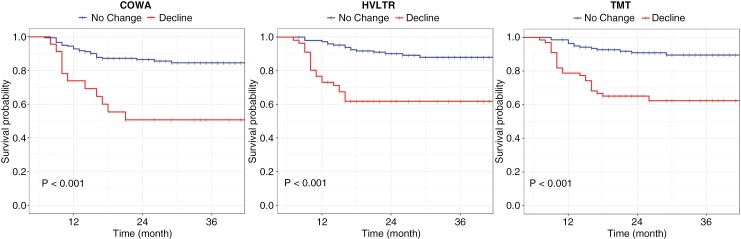
Survival curves for patients with and without neurocognitive decline.

Figure 4 depicts the Kaplan–Meier survival curves of the patients with and without a 6-month decline in COWA, HVLTR, and TMT scores. Log-rank test showed statistically significant lower OS rates for patients with a 6-month decline in the 3 domains (*P* < .001). The median survival time of patients with a 6-month decline in COWA, HVLTR, and TMT was 21 (vs 26), 20 (vs 28), and 22 (vs 28), respectively.

Supplementary Figure 7 shows nomograms of the combined models presenting the risk estimation for 1 sample patient with persistent decline at 3 time points. Transparent Reporting of a multivariable prediction model for Individual Prognosis or Diagnosis (TRIPOD) checklist adhered to ensure that sufficient detail and clarity on the prediction models are provided (Supplementary Table 8).

## Discussion

It is imperative to develop an accurate individualized risk estimation tool to balance the potential advantages of RT against the risk of neurocognitive toxicity for adult patients with a primary brain tumor. In this study, we developed and validated NTCP models using easy-to-obtain clinical and dose–volume predictors to predict the risk of 6-month, 1-year, and 2-year ND after brain irradiation. The NTCP models showed good patient-level predictions with positive net benefits in decision-making.

Our results highlight the low-dose radiosensitivity of the brain tissue. We found that brain *V*_5 Gy_ is a significant risk factor for ND at 6-month and 1-year time points. Moreover, left hippocampus *D*_max_ ≥ 7 Gy showed a significant association with 1-year ND. These results are in line with recent findings from in vitro, in vivo and few clinical studies.^28^ It has been shown that the brain tissue, mainly the regions with more neuronal precursor cells, are exceptionally radiosensitive and therefore more susceptible to neurological damage even at low doses. More specifically, several studies on rat models have shown that low-dose hippocampus irradiation leads to morphological changes as well as decreased cell division and increased inflammation which consequently causes progressive deficits in memory and learning.^11,29^

We found that cerebellum *D*_max_ ≥ 27 Gy played the most significant role in predicting 2-year ND (OR = 2.95). In confirmation of our findings, previous publications have shown that higher dose received by the cerebellum is associated with a decline in several neurocognitive domains, such as educational attainment in children with ependymoma.^14^ Dutz et al. also showed that deterioration of the Montreal Cognitive Assessment (MoCA) score was positively associated with the volume of the anterior cerebellum that received 30–40 Gy.^15^ Dose-dependent cerebellar atrophy also have been observed in glioma patients receiving chemoradiation.^30^ These findings may be explained by complex interactions between the cerebral cortex and the cerebellum. The cerebellum plays a key role in neurocognition such as sensorimotor function and executive function, language and working memory.^31^ Therefore, findings suggest that cerebellum sparing should be considered during RT planning due to its high impact on neurocognition.

In our study, obese and overweight patients were found to be at lower risk of developing ND six months after brain irradiation. An emerging number of recent studies^32–34^ and some isolated historic studies^35,36^ have found that higher BMI is associated with better survival outcomes among patients with different types of cancer. This surprising phenomenon is called “obesity paradox.” The repeated observation of the obesity paradox has encouraged research to find a biological explanation for its occurrence.^37^ One of the hypotheses behind the obesity paradox is the “energy reserve” or “hibernation hypothesis,” which states that excess adipose deposits confer an advantage as a nutrient reserve during anti-cancer treatments (eg surgery, chemotherapy and radiotherapy).^38^ However, we believe that future evidence are required to confirm this finding in the neurocognitive area using body composition metrics that can quantify different body fat components throughout the body.

The positive association between age and ND has been proven by several studies. Wolfson et al. showed that age was a significant predictor for COWA-, HVLTR- and TMT-based decline in patients with Small Cell Lung Cancer (SCLC) who received prophylactic cranial irradiation.^39^ Gondi et al. and Chapman et al. also confirmed that older patients are at higher risk of decline measured by HVLTR delayed recall.^40,41^ Contradictory results are available on the association of education level with the risk of ND. Two studies have shown that higher education is associated with a lower risk of ND in SCLC patients receiving prophylactic cranial irradiation^39^ and patients with intracranial meningioma treated with RT.^42^ However, 1 study found that years of formal education were positively associated with a higher risk of longitudinal RT-related cognitive changes measured by MoCA in patients with nasopharyngeal carcinoma.^43^ The toxic effect of chemotherapy on the decline in MoCA score was confirmed by 1 study in patients with nasopharyngeal carcinoma.^44^ A recent study on a large cohort of glioblastoma patients suggests that an interval of 4–8 weeks between (sub)total resection and RT resulted in better outcomes.^45^

Comparing the net benefits of the clinical, dose–volume and combined models at each time point, we found that clinical variables offered little predictive value for 1-year and 2-year ND. Neurocognitive function as a multifaceted concept has been shown to be affected by several environmental factors, such as family and intimate relationships, social engagement, economic status, career/educational attainments, etc.^46^ Therefore, as time increases, predicting the risk of ND using baseline clinical factors becomes challenging due to synergistic/antagonistic effects of environmental stressors/alleviators. However, it should be noted that accurate prediction of early decline (6 months) takes precedence in our analysis because preliminary clinical trials show that early forms of RT-induced brain damage may be more amenable to neurocognitive rehabilitation therapy.^47^

The discrimination power of the combined NTCP model for 6-month ND was fair, calibration measures showed good to perfect individualized predictions and net benefit was superior for all thresholds compared to the benchmarking lines. Although AUC is a popular statistical measure, it does not take into account the consequence of medical decisions. While AUC considers the entire curve, in practice specific thresholds matter for patient risk stratification. Therefore, it is necessary to take into account calibration and net benefit measures when comparing different models for patient-level decision-making.^27^

Recent evidence suggest that domain-specific neurocognitive impairment may be associated with worse survival in patients with solid tumors.^48^ Executive dysfunction most often reflects difficulties in independent function and interferes with daily responsibilities. Patients with executive disorders may benefit from written instructions and repetition.^49^ On the other hand, patients with working memory impairments often have more difficulty retaining simple educational information, including the treatment guides. Working memory impairments are potentially modifiable by cognitive training strategies.^50^ These deficits can be easily missed or the patients may be labeled as not health literate or unmotivated. Thus, it is crucial to perform regular neurocognitive screening, especially for patients who do not have a family member to provide collateral information. This can help patients to regain their function using relevant cognitive rehabilitation exercises.

The following limitations should be taken into consideration when interpreting our findings. First, although a large proportion of patients agreed to participate in neurocognitive assessments,^21^ refused, potentially introducing participation bias. Second, the sample size and the single-center nature of our study is a limitation. However, considering the low prevalence of primary brain tumor, the sample is rather adequate and includes referrals from several hospitals. Third, due to limited follow-up duration, our findings do not include the ND events which may manifest several years after RT. Fourth, a lack of neurocognitive assessment at the time of diagnosis (and surgery) might have affected the predictive power of the NTCP models.

In conclusion, the developed NTCP models showed good net benefits in decision analysis. Using easy-to-obtain predictors, the models can be used as potentially useful and cost-effective approaches to screen patients who are at high risk of developing neurocognitive dysfunction after brain irradiation. These models have the potential to define radiotherapy planning objectives and select patients for evidence-based post-therapy rehabilitation treatments.

## Supplementary material

Supplementary material is available online at *Neuro-Oncology* (https://academic.oup.com/neuro-oncology).

noae035_suppl_Supplementary_Figures_S1-S7_Tables_S2-S8

## Data Availability

All original data from this manuscript will be made available upon reasonable request.
