# Supplementary material for: Predicting the risk of neurocognitive decline after brain irradiation in adult patients with a primary brain tumor
Source: Neuro Oncol. 2024 Apr 10;26(8):1467–78. doi: 10.1093/neuonc/noae035 (PMC11300005; doi:10.1093/neuonc/noae035)
Supplement: noae035_suppl_Supplementary_Figures_S1-S7_Tables_S2-S8 [file noae035_suppl_supplementary_figures_s1-s7_tables_s2-s8.docx]

**
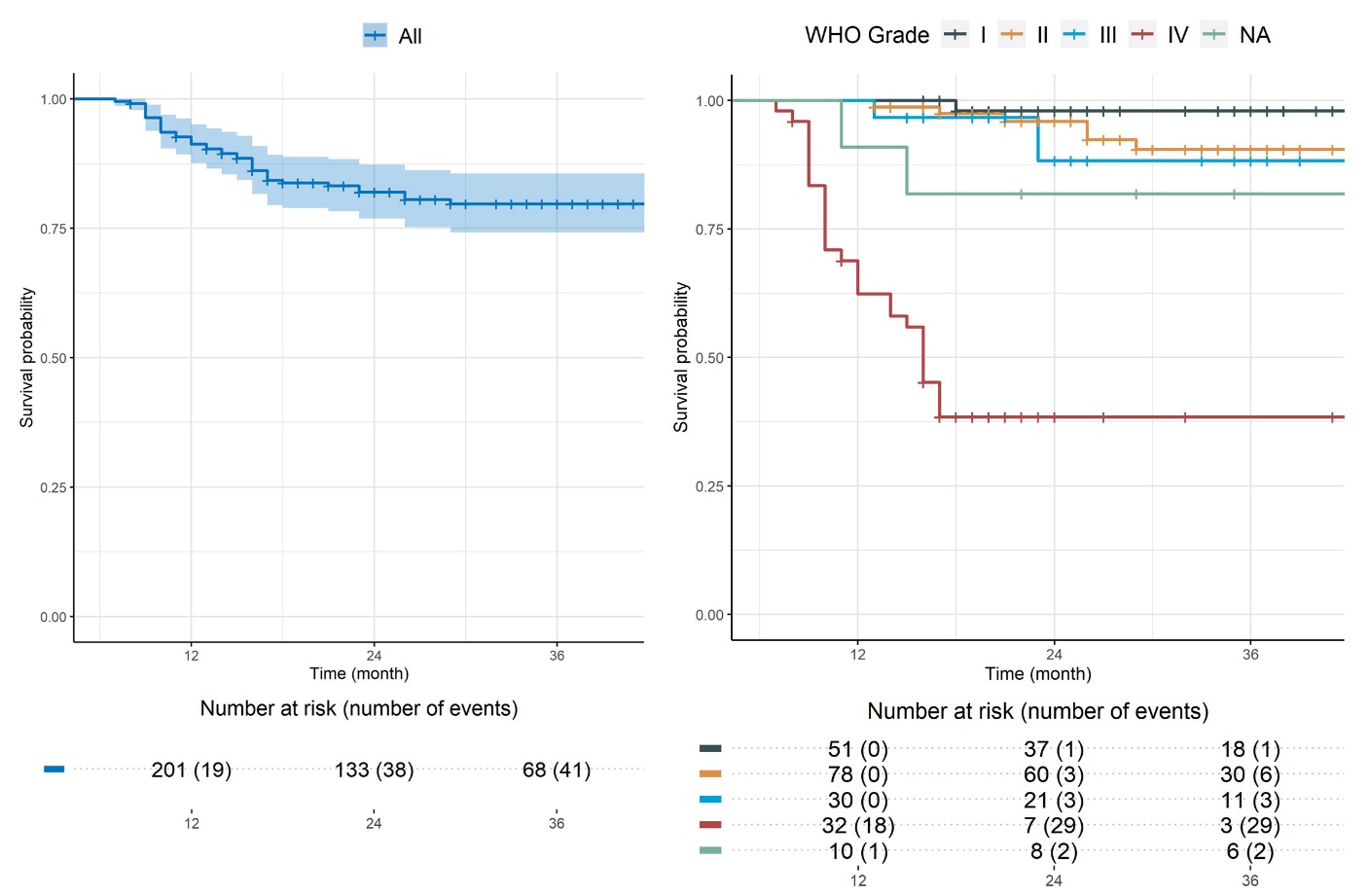
**

**Figure S1.** Kaplan-Meier curves for the overall survival (left) and WHO grade-specific survival (right) for patients with primary brain tumor treated with radiotherapy

**Table S2.** Univariate analysis of clinical characteristics for 6-month, 1-year and 2-year neurocognitive decline in patients with primary brain tumor treated with radiotherapy

| **Variable** | N = 219 | | **6-month** | | | **1-year** | | **2-year** | |
| --- | --- | --- | --- | --- | --- | --- | --- | --- | --- |
|  |  |  | OR (95% CI) | | P | OR (95% CI) | P | OR (95% CI) | P |
| Age at diagnosis |  | |  | |  |  |  |  |  |
| ≤54 | 112 (51%) | | [Reference] | | | [Reference] | | [Reference] | |
| >54 | 107 (49%) | | 4.1 (2.3 to 7.4) | | <0.001 | 1.6 (0.8 to 3.0) | 0.2 | 2.7 (1.2 to 6.3) | 0.020 |
| Age at radiotherapy |  | |  | |  |  |  |  |  |
| ≤56 | 118 (54%) | | [Reference] | | | [Reference] | | [Reference] | |
| >56 | 101 (46%) | | 3.8 (2.2 to 6.9) | | <0.001 | 1.3 (0.7 to 2.4) | 0.5 | 1.9 (0.8 to 4.3) | 0.1 |
| Gender |  | |  | |  |  |  |  |  |
| Female | 117 (53%) | | [Reference] | | | [Reference] | | [Reference] | |
| Male | 102 (47%) | | 0.6 (0.3 to 1.0) | | 0.051 | 0.8 (0.4 to 1.5) | 0.4 | 1.2 (0.6 to 2.7) | 0.6 |
| Weight (kg) | 82 ± 18 | | 0.98 (0.96 to 0.99) | | 0.026 | 0.99 (0.98 to 1.01) | 0.8 | 0.99 (0.97 to 1.01) | 0.4 |
| Height (cm) | 173 ± 10 | | 0.96 (0.93 to 0.99) | | 0.003 | 0.98 (0.95 to 1.01) | 0.2 | 0.98 (0.94 to 1.02) | 0.3 |
| Body mass index |  | |  | |  |  |  |  |  |
| Normal | 65 (30%) | | [Reference] | | | [Reference] | | [Reference] | |
| Overweight | 101 (46%) | | 0.7 (0.4 to 1.1) | | 0.1 | 2.2 (0.2 to 4.7) | 0.4 | 1.0 (0.4 to 2.4) | 0.9 |
| Obese | 53 (24%) | | 0.6 (0.3 to 1.0) | | 0.051 | 1.3 (0.6 to 3.0) | 0.5 | 0.9 (0.3 to 2.5) | 0.9 |
| Education level |  | |  | |  |  |  |  |  |
| Low | 61 (28%) | | [Reference] | | | [Reference] | | [Reference] | |
| Middle | 74 (34%) | | 0.6 (0.3 to 1.3) | | 0.2 | 0.7 (0.3 to 1.6) | 0.4 | 0.3 (0.1 to 0.8) | 0.019 |
| High | 84 (38%) | | 0.4 (0.2 to 0.9) | | 0.024 | 0.5 (0.2 to 1.1) | 0.1 | 0.4 (0.2 to 1.2) | 0.1 |
| Has partner |  | |  | |  |  |  |  |  |
| No | 95 (43%) | | [Reference] | | | [Reference] | | [Reference] | |
| Yes | 124 (57%) | | 1.3 (0.7 to 2.2) | | 0.4 | 1.2 (0.6 to 2.2) | 0.6 | 0.8 (0.4 to 1.7) | 0.6 |
| Living alone |  | |  | |  |  |  |  |  |
| No | 154 (70%) | | [Reference] | | | [Reference] | | [Reference] | |
| Yes | 65 (30%) | | 1.2 (0.7 to 2.2) | | 0.5 | 1.3 (0.6 to 2.6) | 0.5 | 1.3 (0.6 to 3.0) | 0.5 |
| WHO grade |  | |  | |  |  |  |  |  |
| I | 51 (23%) | | [Reference] | | | [Reference] | | [Reference] | |
| II | 78 (36%) | | 0.9 (0.4 to 1.9) | | 0.7 | 1.3 (0.6 to 3.0) | 0.5 | 1.6 (0.5 to 5.8) | 0.5 |
| III | 30 (14%) | | 1.9 (0.7 to 4.9) | | 0.2 | 2.5 (0.9 to 7.4) | 0.091 | NA | NA |
| IV | 49 (22%) | | 10.1 (3.9 to 29.2) | | <0.001 | 2.9 (1.0 to 9.1) | 0.063 | 0.8 (0.1 to 3.7) | 0.7 |
| No grade | 11 (5%) | | 4.1 (1.0 to 21.1) | | 0.060 | 2.4 (0.5 to 11.2) | 0.2 | 0.7 (0.3 to 1.5) | 0.3 |
| Laterality |  | |  | |  |  |  |  |  |
| Left | 88 (40%) | | [Reference] | | | [Reference] | | [Reference] | |
| Right | 111 (51%) | | 1.0 (0.6 to 1.8) | | 0.9 | 1.0 (0.5 to 1.8) | 0.9 | 0.4 (0.1 to 1.5) | 0.2 |
| Midline | 20 (9.1%) | | 0.3 (0.1 to 0.9) | | 0.037 | NA | NA | 3.3 (0.9 to 13.5) | 0.073 |
| Location |  | |  | |  |  |  |  |  |
| Frontal | 82 (37%) | | [Reference] | | | [Reference] | | [Reference] | |
| Temporal | 45 (21%) | | 1.3 (0.6 to 2.7) | | 0.5 | 2.4 (1.0 to 6.3) | 0.058 | 1.1 (0.3 to 3.9) | 0.9 |
| Parietal | 29 (13%) | | 1.3 (0.5 to 3.2) | | 0.6 | 1.7 (0.7 to 4.6) | 0.3 | 0.5 (0.1 to 1.6) | 0.3 |
| Base of skull | 22 (10%) | | 0.5 (0.2 to 1.4) | | 0.2 | 0.5 (0.2 to 1.5) | 0.2 | 0.5 (0.2 to 1.8) | 0.3 |
| Other | 41 (19%) | | 0.7 (0.3 to 1.6) | | 0.5 | 0.9 (0.3 to 2.2) | 0.8 | NA | NA |
| Histology |  | |  | |  |  |  |  |  |
| Meningioma | 56 (26%) | | [Reference] | | | [Reference] | | [Reference] | |
| Glioblastoma | 49 (22%) | | 6.7 (2.6 to 18.8) | | <0.001 | 2.0 (0.7 to 6.0) | 0.2 | 0.6 (0.2 to 1.8) | 0.4 |
| Astrocytoma | 43 (20%) | | 0.6 (0.3 to 1.4) | | 0.3 | 0.8 (0.3 to 2.1) | 0.7 | 1.2 (0.4 to 3.5) | 0.7 |
| Oligodendroglioma | 35 (16%) | | 1.0 (0.4 to 2.5) | | 0.9 | 1.1 (0.4 to 2.7) | 0.8 | 0.8 (0.2 to 2.4) | 0.6 |
| Other | 36 (16%) | | 0.5 (0.2 to 1.1) | | 0.1 | 0.9 (0.4 to 2.4) | 0.9 | 2.2 (0.2 to 23.2) | 0.5 |
| Surgery |  | |  | |  |  |  |  |  |
| None | 30 (14%) | | [Reference] | | | [Reference] | | [Reference] | |
| Biopsy | 24 (11%) | | 1.4 (0.4 to 4.5) | | 0.6 | 2.4 (0.6 to 9.4) | 0.2 | 1.8 (0.6 to 6.0) | 0.3 |
| Resection | 165 (75%) | | 0.7 (0.3 to 1.6) | | 0.4 | 1.4 (0.6 to 3.6) | 0.4 | 1.9 (0.6 to 6.5) | 0.3 |
| Number of surgeries |  | |  | |  |  |  |  |  |
| 0 | 30 (14%) | | [Reference] | | | [Reference] | | [Reference] | |
| 1 | 152 (69%) | | 0.8 (0.4 to 1.9) | | 0.6 | 1.6 (0.7 to 4.1) | 0.3 | 1.5 (0.4 to 6.2) | 0.5 |
| 2-3 | 37 (17%) | | 0.6 (0.2 to 1.6) | | 0.3 | 1.2 (0.4 to 3.6) | 0.8 | 0.4 (0.0 to 5.0) | 0.5 |
| Surgery to radiotherapy |  | |  | |  |  |  |  |  |
| 1-2 months | 87 (40%) | | [Reference] | | | [Reference] | | [Reference] | |
| 3-6 months | 48 (22%) | | 0.3 (0.1 to 0.6) | | 0.001 | 0.3 (0.1 to 0.7) | 0.008 | 0.2 (0.0 to 2.6) | 0.2 |
| ≥7 months | 54 (25%) | | 0.3 (0.1 to 0.5) | | <0.001 | 0.6 (0.3 to 1.3) | 0.2 | 0.6 (0.0 to 6.6) | 0.7 |
| No surgery | 30 (14%) | | 0.6 (0.3 to 1.6) | | 0.3 | 0.4 (0.1 to 1.0) | 0.067 | 0.2 (0.0 to 2.9) | 0.3 |
| Chemotherapy |  | |  | |  |  |  |  |  |
| No | 94 (43%) | | [Reference] | | | [Reference] | | [Reference] | |
| Yes | 125 (57%) | | 2.2 (1.3 to 4.0) | | 0.005 | 1.3 (0.7 to 2.4) | 0.5 | 0.9 (0.4 to 2.0) | 0.8 |
| Chemotherapy agent |  | |  | |  |  |  |  |  |
| None | 94 (43%) | | [Reference] | | | [Reference] | | [Reference] | |
| PCV | 34 (16%) | | 1.3 (0.6 to 3.0) | | 0.5 | 1.2 (0.5 to 2.7) | 0.7 | 1.4 (0.5 to 3.5) | 0.5 |
| TMZ | 91 (42%) | | 2.7 (1.5 to 5.1) | | 0.001 | 1.3 (0.7 to 2.7) | 0.4 | 0.6 (0.2 to 1.5) | 0.3 |
| Diagnosis to radiotherapy |  |  | |  | |  |  |  |  |
| 1 month | 107 (49%) | [Reference] | | | | [Reference] | | [Reference] | |
| 2-3 months | 60 (27%) | 0.8 (0.4 to 1.4) | | 0.4 | | 0.7 (0.3 to 1.5) | 0.3 | 0.5 (0.2 to 1.4) | 0.2 |
| ≥4 months | 52 (24%) | 0.7 (0.3 to 1.3) | | 0.2 | | 0.4 (0.2 to 0.8) | 0.015 | 0.3 (0.1 to 0.9) | 0.033 |
| Modality of radiotherapy |  |  | |  | |  |  |  |  |
| Photon | 118 (54%) | [Reference] | | | | [Reference] | | [Reference] | |
| Proton | 24 (11%) | 0.4 (0.2 to 1.1) | | 0.079 | | 0.5 (0.2 to 1.4) | 0.2 | 3.1 (0.7 to 16.5) | 0.1 |
| Photon and proton | 77 (35%) | 0.4 (0.2 to 0.8) | | 0.007 | | 1.0 (0.5 to 2.0) | 0.9 | 1.1 (0.5 to 2.6) | 0.8 |
| Prescribed dose (Gy) |  |  | |  | |  |  |  |  |
| 40.05-46.8 | 22 (10%) | [Reference] | | | | [Reference] | | [Reference] | |
| 50.4-52.2 | 88 (40%) | 0.4 (0.1 to 1.0) | | 0.056 | | 0.6 (0.2 to 2.0) | 0.4 | 3.3 (0.5 to 65.2) | 0.3 |
| 54-59.4 | 71 (32%) | 0.5 (0.2 to 1.3) | | 0.2 | | 0.9 (0.3 to 3.1) | 0.9 | 5.8 (0.8 to 10.2) | 0.1 |
| 60 | 38 (17%) | 2.6 (0.8 to 9.1) | | 0.1 | | 1.1 (0.3 to 5.0) | 0.9 | NA | NA |
| Fraction dose (Gy) |  |  | |  | |  |  |  |  |
| 1.8 | 170 (78%) | [Reference] | | | | [Reference] | | [Reference] | |
| 2 | 38 (17%) | 6.5 (2.8 to 16.8) | | <0.001 | | 1.6 (0.5 to 4.7) | 0.4 | NA | NA |
| 2.67 | 11 (5%) | NA | | NA | | NA | NA | 1.4 (0.6 to 3.1) | 0.4 |
| Radiotherapy duration (day) |  |  | |  | |  |  |  |  |
| <40 | 109 (50%) | [Reference] | | | | [Reference] | | [Reference] | |
| ≥40 | 110 (50%) | 1.5 (0.9 to 2.6) | | 0.2 | | 1.4 (0.7 to 2.6) | 0.3 | 0.9 (0.3 to 2.2) | 0.8 |
| Diabetes |  |  | |  | |  |  |  |  |
| None | 197 (90%) | [Reference] | | | | [Reference] | | [Reference] | |
| Yes | 22 (10%) | 1.0 (0.4 to 2.4) | | 0.9 | | 1.3 (0.4 to 3.9) | 0.7 | 2.4 (0.6 to 12.2) | 0.3 |
| Hypertension |  |  | |  | |  |  |  |  |
| No | 145 (66%) | [Reference] | | | | [Reference] | | [Reference] | |
| Yes | 74 (34%) | 1.3 (0.7 to 2.3) | | 0.4 | | 1.3 (0.7 to 2.6) | 0.4 | 1.7 (0.7 to 4.0) | 0.2 |
| Hyperlipidaemia |  |  | |  | |  |  |  |  |
| No | 172 (79%) | [Reference] | | | | [Reference] | | [Reference] | |
| Yes | 47 (21%) | 1.2 (0.6 to 2.3) | | 0.6 | | 1.2 (0.6 to 2.7) | 0.6 | 1.4 (0.5 to 4.0) | 0.5 |
| Cardiac diseases |  |  | |  | |  |  |  |  |
| No | 185 (84%) | [Reference] | | | | [Reference] | | [Reference] | |
| Yes | 34 (16%) | 1.2 (0.5 to 2.5) | | 0.7 | | 0.7 (0.3 to 1.6) | 0.4 | 1.0 (0.3 to 2.6) | 0.9 |
| Cerebrovascular diseases |  |  | |  | |  |  |  |  |
| No | 190 (87%) | [Reference] | | | | [Reference] | | [Reference] | |
| Yes | 29 (13%) | 2.4 (1.0 to 5.7) | | 0.046 | | 1.8 (0.6 to 5.6) | 0.3 | 1.4 (0.3 to 6.1) | 0.7 |
| Psychological diseases |  |  | |  | |  |  |  |  |
| No | 186 (85%) | [Reference] | | | | [Reference] | | [Reference] | |
| Yes | 33 (15%) | 1.5 (0.7 to 3.2) | | 0.3 | | 2.5 (1.0 to 6.6) | 0.057 | 0.9 (0.3 to 2.6) | 0.8 |
| Respiratory diseases |  |  | |  | |  |  |  |  |
| No | 183 (84%) | [Reference] | | | | [Reference] | | [Reference] | |
| Yes | 36 (16%) | 1.4 (0.7 to 3.1) | | 0.3 | | 1.0 (0.4 to 2.3) | 0.9 | 0.7 (0.2 to 2.0) | 0.5 |
| Autoimmune diseases |  |  | |  | |  |  |  |  |
| No | 202 (92%) | [Reference] | | | | [Reference] | | [Reference] | |
| Yes | 17 (7.8%) | 1.2 (0.4 to 3.4) | | 0.8 | | 1.8 (0.6 to 6.4) | 0.3 | 1.1 (0.3 to 4.3) | 0.9 |
| Other neoplasms |  |  | |  | |  |  |  |  |
| No | 193 (88%) | [Reference] | | | | [Reference] | | [Reference] | |
| Yes | 26 (12%) | 1.7 (0.7 to 4.1) | | 0.2 | | 0.9 (0.3 to 2.8) | 0.9 | 0.9 (0.2 to 3.3) | 0.9 |
| Thyroid disorders |  |  | |  | |  |  |  |  |
| No | 192 (88%) | [Reference] | | | | [Reference] | | [Reference] | |
| Yes | 27 (12%) | 0.5 (0.2 to 1.1) | | 0.098 | | 1.2 (0.5 to 2.9) | 0.8 | 1.0 (0.3 to 3.2) | 0.9 |
| Ocular malfunctions |  |  | |  | |  |  |  |  |
| No | 185 (84%) | [Reference] | | | | [Reference] | | [Reference] | |
| Yes | 34 (16%) | 1.4 (0.7 to 3.1) | | 0.3 | | 0.8 (0.3 to 2.1) | 0.6 | 1.6 (0.6 to 4.5) | 0.3 |
| Smoking |  |  | |  | |  |  |  |  |
| None | 120 (55%) | [Reference] | | | | [Reference] | | [Reference] | |
| Current | 23 (11%) | 1.3 (0.5 to 3.4) | | 0.6 | | 1.5 (0.5 to 4.4) | 0.5 | 3.5 (1.1 to 12.6) | 0.040 |
| Former | 76 (35%) | 0.8 (0.5 to 1.5) | | 0.6 | | 1.4 (0.7 to 2.8) | 0.3 | 1.3 (0.5 to 3.3) | 0.5 |
| CCI |  |  | |  | |  |  |  |  |
| 2-3 | 120 (55%) | [Reference] | | | | [Reference] | | [Reference] | |
| ≥4 | 99 (45%) | 2.8 (1.6 to 5.0) | | <0.001 | | 1.3 (0.7 to 2.5) | 0.4 | 2.2 (1.0 to 5.3) | 0.065 |
| KPS (%) |  |  | |  | |  |  |  |  |
| 90-100 | 132 (60%) | [Reference] | | | | [Reference] | | [Reference] | |
| 70-80 | 87 (40%) | 1.4 (0.8 to 2.6) | | 0.2 | | 1.6 (0.8 to 3.0) | 0.2 | 1.3 (0.6 to 3.0) | 0.5 |
| Antithrombotic |  |  | |  | |  |  |  |  |
| No | 199 (91%) | [Reference] | | | | [Reference] | | [Reference] | |
| Yes | 20 (9.1%) | 1.6 (0.6 to 4.2) | | 0.4 | | 0.5 (0.1 to 1.9) | 0.4 | 0.7 (0.1 to 3.5) | 0.6 |
| Antiepileptic |  |  | |  | |  |  |  |  |
| No | 138 (63%) | [Reference] | | | | [Reference] | | [Reference] | |
| Yes | 81 (37%) | 1.3 (0.7 to 2.3) | | 0.4 | | 1.1 (0.6 to 2.0) | 0.8 | 0.9 (0.4 to 1.9) | 0.7 |
| Sleep medications |  |  | |  | |  |  |  |  |
| No | 186 (85%) | [Reference] | | | | [Reference] | | [Reference] | |
| Yes | 33 (15%) | 0.5 (0.2 to 1.2) | | 0.1 | | 1.9 (0.8 to 4.3) | 0.1 | 2.3 (0.8 to 7.3) | 0.2 |
| Psychological medications |  |  | |  | |  |  |  |  |
| No | 184 (84%) | [Reference] | | | | [Reference] | | [Reference] | |
| Yes | 35 (16%) | 1.5 (0.7 to 3.3) | | 0.3 | | 1.2 (0.5 to 2.8) | 0.7 | 1.1 (0.3 to 3.1) | 0.9 |
| Vitamin D |  |  | |  | |  |  |  |  |
| No | 185 (84%) | [Reference] | | | | [Reference] | | [Reference] | |
| Yes | 34 (16%) | 1.2 (0.6 to 2.4) | | 0.7 | | 1.3 (0.6 to 3.0) | 0.5 | 1.9 (0.7 to 5.8) | 0.2 |
| Antidiabetic |  |  | |  | |  |  |  |  |
| No | 198 (90%) | [Reference] | | | | [Reference] | | [Reference] | |
| Yes | 21 (9.6%) | 0.9 (0.4 to 2.2) | | 0.8 | | 1.3 (0.4 to 3.9) | 0.7 | 2.4 (0.6 to 12.2) | 0.3 |
| Antihypertensive |  |  | |  | |  |  |  |  |
| No | 149 (68%) | [Reference] | | | | [Reference] | | [Reference] | |
| Yes | 70 (32%) | 1.2 (0.7 to 2.1) | | 0.6 | | 1.2 (0.6 to 2.4) | 0.5 | 1.5 (0.6 to 3.6) | 0.3 |
| Statin |  |  | |  | |  |  |  |  |
| No | 173 (79%) | [Reference] | | | | [Reference] | | [Reference] | |
| Yes | 46 (21%) | 1.1 (0.6 to 2.2) | | 0.7 | | 1.2 (0.6 to 2.7) | 0.6 | 1.4 (0.5 to 4.0) | 0.5 |
| Steroid |  |  | |  | |  |  |  |  |
| No | 170 (78%) | [Reference] | | | | [Reference] | | [Reference] | |
| Yes | 49 (22%) | 2.2 (1.1 to 4.5) | | 0.021 | | 1.7 (0.8 to 3.9) | 0.2 | 3.1 (0.9 to 12.1) | 0.085 |
| CTCAE Seizure |  |  | |  | |  |  |  |  |
| 0 | 167 (76%) | [Reference] | | | | [Reference] | | [Reference] | |
| 1-3 | 52 (24%) | 1.7 (0.9 to 3.3) | | 0.1 | | 1.6 (0.8 to 3.3) | 0.2 | 2.0 (0.9 to 4.9) | 0.1 |
| CTCAE Amnesia |  |  | |  | |  |  |  |  |
| 0 | 160 (73%) | [Reference] | | | | [Reference] | | [Reference] | |
| 1-2 | 59 (27%) | 1.1 (0.6 to 2.0) | | 0.9 | | 1.3 (0.6 to 2.6) | 0.5 | 1.8 (0.8 to 4.4) | 0.2 |
| CTCAE Dizziness |  |  | |  | |  |  |  |  |
| 0 | 197 (90%) | [Reference] | | | | [Reference] | | [Reference] | |
| 1-2 | 22 (10%) | 1.3 (0.5 to 3.5) | | 0.6 | | 0.7 (0.2 to 2.0) | 0.5 | 1.0 (0.3 to 3.2) | 0.9 |
| CTCAE Headache |  |  | |  | |  |  |  |  |
| 0 | 177 (81%) | [Reference] | | | | [Reference] | | [Reference] | |
| 1 | 42 (19%) | 0.8 (0.4 to 1.7) | | 0.6 | | 0.9 (0.4 to 2.1) | 0.9 | 0.7 (0.3 to 1.9) | 0.5 |
| Baseline COWA total words |  |  | |  | |  |  |  |  |
| ≤25 | 56 (26%) | 1.6 (0.7 to 3.4) | | 0.2 | | 2.0 (0.8 to 5.0) | 0.1 | 0.9 (0.3 to 2.6) | 0.8 |
| 26-41 | 107 (49%) | 1.9 (1.0 to 3.7) | | 0.072 | | 2.8 (1.3 to 6.5) | 0.011 | 1.0 (0.4 to 2.6) | 0.9 |
| ≥42 | 56 (26%) | [Reference] | | | | [Reference] | | [Reference] | |
| Baseline HVLTR free recall three trials |  |  | |  | |  |  |  |  |
| ≤21 | 59 (27%) | 2.2 (1.1 to 4.8) | | 0.036 | | 2.1 (0.9 to 5.2) | 0.085 | 3.3 (1.1 to 11.0) | 0.042 |
| 22-29 | 97 (44%) | 1.8 (0.9 to 3.4) | | 0.089 | | 1.7 (0.8 to 3.7) | 0.2 | 1.2 (0.5 to 3.1) | 0.7 |
| 30-36 | 63 (29%) | [Reference] | | | | [Reference] | | [Reference] | |
| Baseline HVLTR delayed recall correct answers |  |  | |  | |  |  |  |  |
| ≤6 | 55 (25%) | 1.9 (0.9 to 4.1) | | 0.1 | | 1.9 (0.8 to 4.6) | 0.1 | 5.3 (1.6 to 18.8) | 0.007 |
| 7-10 | 98 (45%) | 1.3 (0.7 to 2.4) | | 0.5 | | 1.6 (0.8 to 3.5) | 0.2 | 1.2 (0.5 to 3.3) | 0.6 |
| 11-12 | 66 (30%) | [Reference] | | | | [Reference] | | [Reference] | |
| Baseline HVLTR delayed recognition total TP |  |  | |  | |  |  |  |  |
| ≤11 | 86 (39%) | 1.0 (0.6 to 1.8) | | 0.9 | | 1.6 (0.8 to 3.0) | 0.2 | 1.6 (0.7 to 3.6) | 0.3 |
| 12 | 133 (61%) | [Reference] | | | | [Reference] | | [Reference] | |
| Baseline HVLTR delayed recognition related FP |  |  | |  | |  |  |  |  |
| No | 142 (65%) | [Reference] | | | | [Reference] | | [Reference] | |
| Yes | 77 (35%) | 1.8 (1.0 to 3.3) | | 0.042 | | 0.9 (0.4 to 1.7) | 0.7 | 1.7 (0.8 to 3.9) | 0.2 |
| Baseline HVLTR delayed recognition unrelated FP |  |  | |  | |  |  |  |  |
| No | 193 (88%) | [Reference] | | | | [Reference] | | [Reference] | |
| Yes | 26 (12%) | 0.8 (0.3 to 2.0) | | 0.7 | | 1.1 (0.4 to 3.1) | 0.8 | 0.5 (0.1 to 2.1) | 0.4 |
| Baseline TMT part A time (sec) |  |  | |  | |  |  |  |  |
| ≤32 | 100 (46%) | [Reference] | | | | [Reference] | | [Reference] | |
| >32 | 119 (54%) | 2.3 (1.3 to 4.1) | | 0.004 | | 1.8 (1.0 to 3.4) | 0.065 | 2.7 (1.2 to 6.2) | 0.014 |
| Baseline TMT part B time (sec) |  |  | |  | |  |  |  |  |
| ≤77 | 112 (51%) | [Reference] | | | | [Reference] | | [Reference] | |
| >77 | 107 (49%) | 2.9 (1.7 to 5.2) | | <0.001 | | 1.8 (1.0 to 3.4) | 0.061 | 3.6 (1.6 to 8.4) | 0.002 |

**Abbreviations:** CCI, Charlson Comorbidity Index; CI, Confidence Interval; COWA, Controlled Oral Word Association Test; CTCAE, Common Terminology Criteria for Adverse Events; FP, False Positive; HVLTR, Hopkins Verbal Learning Test Revised; KPS, Karnofsky Performance Scale; OR, Odds Ratio; PCV, Procarbazine, Lomustine (CCNU) and Vincristine; TMT, Trail Making Test; TMZ, Temozolomide; TP, True Positive; WHO, World Health Organization

**Table S3.** Clinical, dose-volume and combined models for predicting the risk of 1-year neurocognitive decline in patients with primary brain tumor treated with radiotherapy

| **Clinical Model** | **OR (95% CI)** | **P** | **Combined Model** | **OR (95% CI)** | **P** |
| --- | --- | --- | --- | --- | --- |
| (Intercept) | 1.72 (0.73 to 4.02) | 0.2 | (Intercept) | 0.39 (0.13 to 1.21) | 0.1 |
| Tumor location |  |  | Time between surgery and radiotherapy (month) |  | |
| Frontal | [Reference] | | 1-2 | [Reference] | |
| Temporal | 2.62 (1.01 to 6.83) | 0.049 | 3-6 | 0.42 (0.17 to 1.07) | 0.069 |
| Parietal | 1.45 (0.52 to 4.07) | 0.5 | ≥7 | 1.49 (0.5 to 4.45) | 0.5 |
| Base of skull | 0.44 (0.12 to 1.66) | 0.2 | No surgery | 1.67 (0.43 to 6.51) | 0.5 |
| Other | 1.03 (0.37 to 2.85) | 0.9 | Brain D_mean_ (≥10Gy vs <10Gy) | 5.54 (2.04 to 8.04) | 0.001 |
| Education level |  | | Left hippocampus D_max_ (≥7Gy vs <7Gy) | 1.98 (0.97 to 4.05) | 0.06 |
| Low | [Reference] | | Brainstem interior volume (≥16cc vs <16cc) | 0.36 (0.18 to 0.74) | 0.006 |
| Middle | 0.69 (0.3 to 1.61) | 0.4 |  |  |  |
| High | 0.46 (0.2 to 1.06) | 0.067 |  |  |  |
| Time between surgery and radiotherapy (month) |  | |  |  |  |
| 1-2 | [Reference] | |  |  |  |
| 3-6 | 0.32 (0.13 to 0.77) | 0.011 |  |  |  |
| ≥7 | 0.69 (0.28 to 1.68) | 0.4 |  |  |  |
| No surgery | 0.59 (0.18 to 1.93) | 0.4 |  |  |  |
| **Dose-volume Model** | **OR (95% CI)** | **P** |  |  |  |
| (Intercept) | 0.47 (0.24 to 0.92) | 0.029 |  |  |  |
| Brain D_mean_ (≥10Gy vs <10Gy) | 3.56 (1.78 to 7.14) | <0.001 |  |  |  |
| Left hippocampus D_max_ (≥7Gy vs <7Gy) | 2.05 (1.03 to 4.09) | 0.041 |  |  |  |
| Brainstem interior volume (≥16cc vs <16cc) | 0.37 (0.18 to 0.74) | 0.005 |  |  |  |


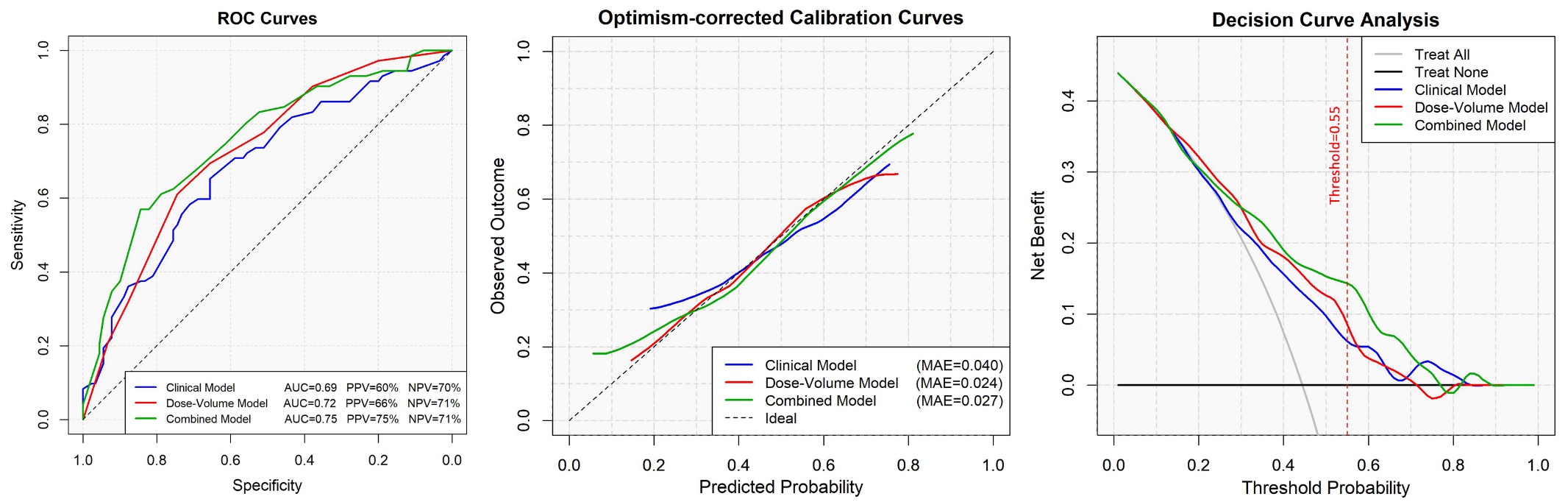


**Figure S4.** The Receiver Operating Characteristic (ROC) curves, calibration plot and decision curve analysis of the clinical, dose-volume and combined models for predicting the risk of 1-year neurocognitive decline in patients with primary brain tumor treated with radiotherapy. The optimal threshold was determined using the Youden index method.

**Table S5.** Clinical, dose-volume and combined models for predicting the risk of 2-year neurocognitive decline in patients with primary brain tumor treated with radiotherapy

| **Clinical Model** | **OR (95% CI)** | **P** | **Combined Model** | **OR (95% CI)** | **P** |
| --- | --- | --- | --- | --- | --- |
| (Intercept) | 0.88 (0.34 to 2.25) | 0.8 | (Intercept) | 0.13 (0.04 to 0.38) | <0.001 |
| Education level |  |  | TMT part A time (sec) |  |  |
| Low | [Reference] | | ≤32 | [Reference] | |
| Middle | 0.34 (0.12 to 1.02) | 0.055 | >32 | 2.66 (1.13 to 6.25) | 0.025 |
| High | 0.52 (0.19 to 1.42) | 0.2 | Brain D_max_ (≥54Gy vs <54Gy) | 2.91 (1.11 to 7.61) | 0.03 |
| TMT part A time (sec) |  |  | Cerebellum D_max_ (≥27Gy vs <27Gy) | 2.95 (1.25 to 6.96) | 0.014 |
| ≤32 | [Reference] |  |  |  |  |
| >32 | 2.36 (1.03 to 5.41) | 0.044 |  |  |  |
| **Dose-volume Model** | **OR (95% CI)** | **P** |  |  |  |
| (Intercept) | 0.21 (0.08 to 0.54) | 0.001 |  |  |  |
| Brain D_max_ (≥54Gy vs <54Gy) | 2.9 (1.14 to 7.39) | 0.026 |  |  |  |
| Cerebellum D_max_ (≥27Gy vs <27Gy) | 3.02 (1.31 to 6.97) | 0.01 |  |  |  |

**
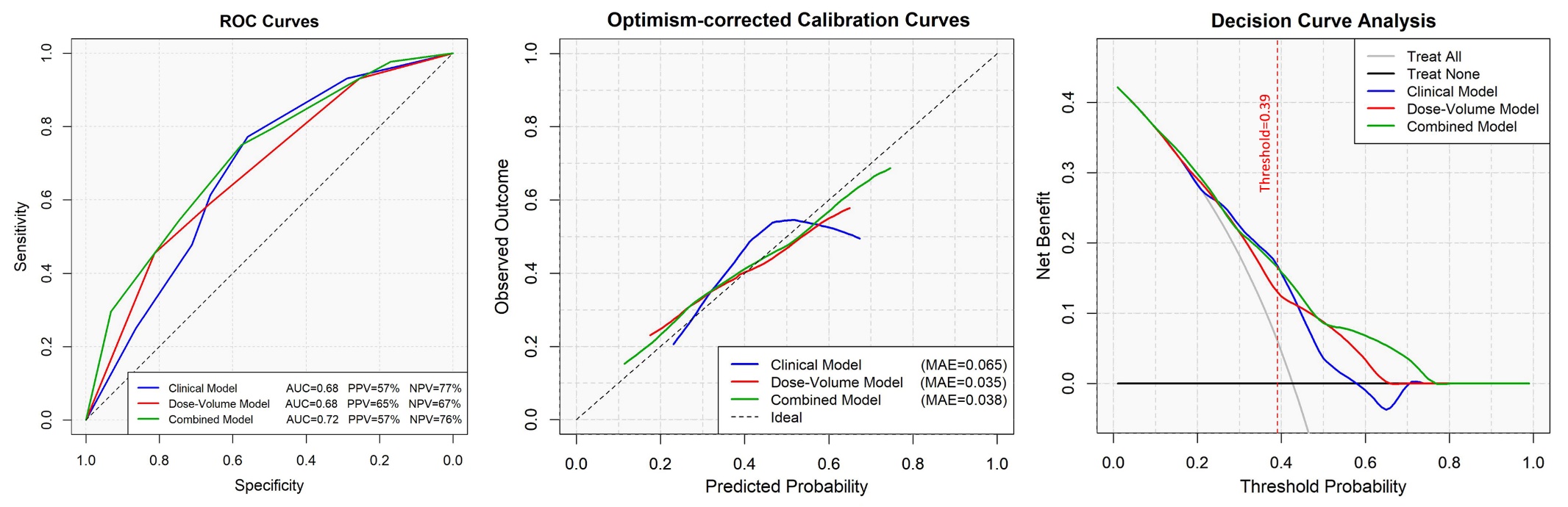
**

**Figure S6.** The Receiver Operating Characteristic (ROC) curves, calibration plot and decision curve analysis of the clinical, dose-volume and combined models for predicting the risk of 2-year neurocognitive decline in patients with primary brain tumor treated with radiotherapy. The optimal threshold was determined using the Youden index method.

**
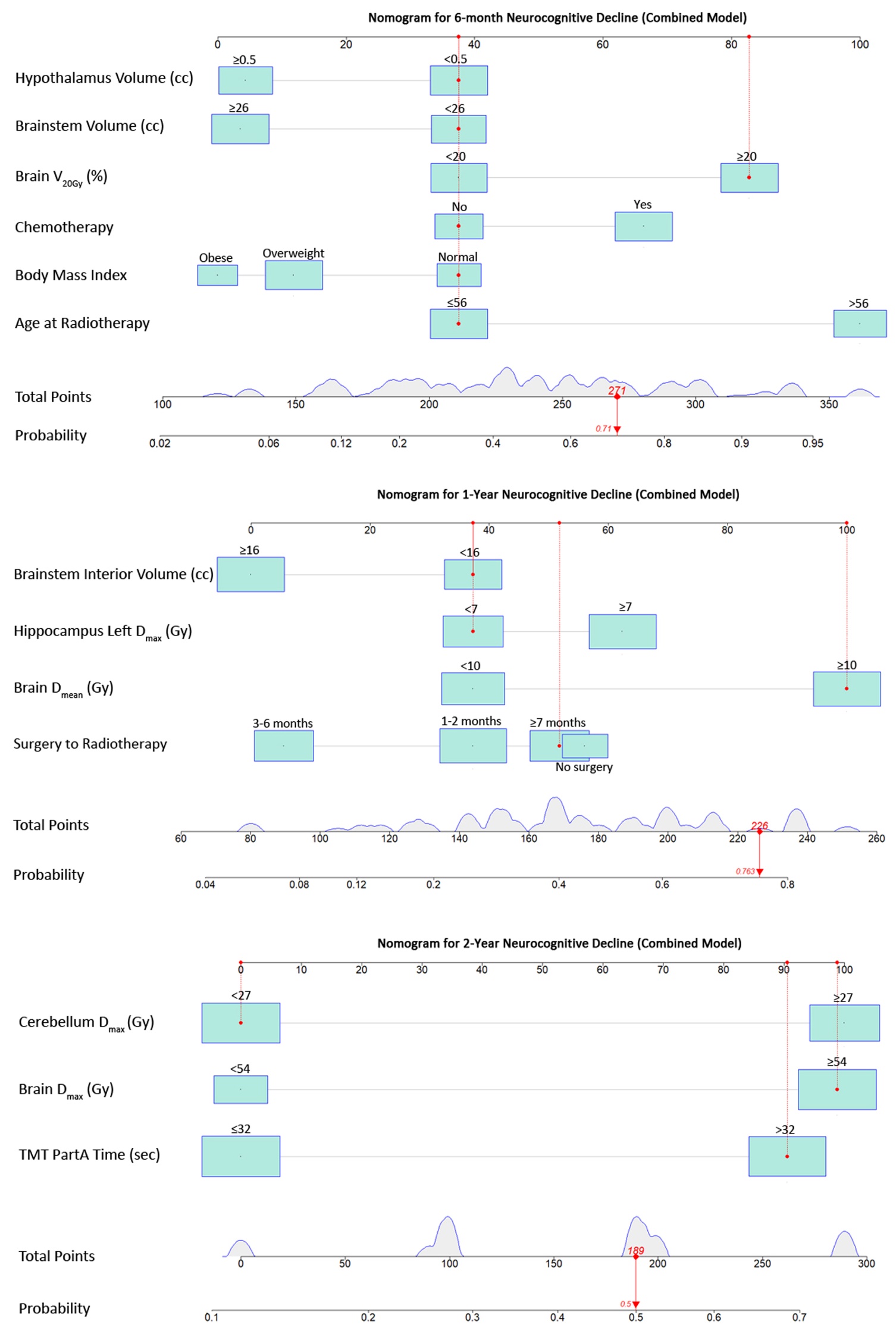
Figure S7.** Nomograms of the combined models for 6-month, 1-year and 2-year neurocognitive decline

**Table S8.** TRIPOD checklist for prediction model development and validation

| **Section/Topic** | **Item** |  | **Checklist Item** | **Page** |
| --- | --- | --- | --- | --- |
| **Title and abstract** | | | | |
| Title | 1 | D;V | Identify the study as developing and/or validating a multivariable prediction model, the target population, and the outcome to be predicted. | 1 |
| Abstract | 2 | D;V | Provide a summary of objectives, study design, setting, participants, sample size, predictors, outcome, statistical analysis, results, and conclusions. | 2 |
| **Introduction** | | | | |
| Background and objectives | 3a | D;V | Explain the medical context (including whether diagnostic or prognostic) and rationale for developing or validating the multivariable prediction model, including references to existing models. | 4 |
|  | 3b | D;V | Specify the objectives, including whether the study describes the development or validation of the model or both. | 4 |
| **Methods** | | | | |
| Source of data | 4a | D;V | Describe the study design or source of data (e.g., randomized trial, cohort, or registry data), separately for the development and validation data sets, if applicable. | 5 |
|  | 4b | D;V | Specify the key study dates, including start of accrual; end of accrual; and, if applicable, end of follow-up. | 5 |
| Participants | 5a | D;V | Specify key elements of the study setting (e.g., primary care, secondary care, general population) including number and location of centres. | 5 |
|  | 5b | D;V | Describe eligibility criteria for participants. | 5 |
|  | 5c | D;V | Give details of treatments received, if relevant. | 5 |
| Outcome | 6a | D;V | Clearly define the outcome that is predicted by the prediction model, including how and when assessed. | 6 |
|  | 6b | D;V | Report any actions to blind assessment of the outcome to be predicted. | 6 |
| Predictors | 7a | D;V | Clearly define all predictors used in developing or validating the multivariable prediction model, including how and when they were measured. | 5-6 |
|  | 7b | D;V | Report any actions to blind assessment of predictors for the outcome and other predictors. | 5-6 |
| Sample size | 8 | D;V | Explain how the study size was arrived at. | 5 |
| Missing data | 9 | D;V | Describe how missing data were handled (e.g., complete-case analysis, single imputation, multiple imputation) with details of any imputation method. | 6 |
| Statistical analysis methods | 10a | D | Describe how predictors were handled in the analyses. | 5-6 |
|  | 10b | D | Specify type of model, all model-building procedures (including any predictor selection), and method for internal validation. | 6-7 |
|  | 10c | V | For validation, describe how the predictions were calculated. | NA |
|  | 10d | D;V | Specify all measures used to assess model performance and, if relevant, to compare multiple models. | 6-7 |
|  | 10e | V | Describe any model updating (e.g., recalibration) arising from the validation, if done. | NA |
| Risk groups | 11 | D;V | Provide details on how risk groups were created, if done. | 6 |
| Development vs. validation | 12 | V | For validation, identify any differences from the development data in setting, eligibility criteria, outcome, and predictors. | NA |
| **Results** | | | | |
| Participants | 13a | D;V | Describe the flow of participants through the study, including the number of participants with and without the outcome and, if applicable, a summary of the follow-up time. A diagram may be helpful. | 8 |
|  | 13b | D;V | Describe the characteristics of the participants (basic demographics, clinical features, available predictors), including the number of participants with missing data for predictors and outcome. | 8-Table 1 |
|  | 13c | V | For validation, show a comparison with the development data of the distribution of important variables (demographics, predictors and outcome). | NA |
| Model development | 14a | D | Specify the number of participants and outcome events in each analysis. | 8 |
|  | 14b | D | If done, report the unadjusted association between each candidate predictor and outcome. | Table S2 |
| Model specification | 15a | D | Present the full prediction model to allow predictions for individuals (i.e., all regression coefficients, and model intercept or baseline survival at a given time point). | Table 2, S3, S5 |
|  | 15b | D | Explain how to the use the prediction model. | 9 |
| Model performance | 16 | D;V | Report performance measures (with CIs) for the prediction model. | 9 |
| Model-updating | 17 | V | If done, report the results from any model updating (i.e., model specification, model performance). | NA |
| **Discussion** | | | | |
| Limitations | 18 | D;V | Discuss any limitations of the study (such as nonrepresentative sample, few events per predictor, missing data). | 11 |
| Interpretation | 19a | V | For validation, discuss the results with reference to performance in the development data, and any other validation data. | NA |
|  | 19b | D;V | Give an overall interpretation of the results, considering objectives, limitations, results from similar studies, and other relevant evidence. | 10-12 |
| Implications | 20 | D;V | Discuss the potential clinical use of the model and implications for future research. | 11-12 |
| **Other information** | | | | |
| Supplementary information | 21 | D;V | Provide information about the availability of supplementary resources, such as study protocol, Web calculator, and data sets. | Figure S7 |
| Funding | 22 | D;V | Give the source of funding and the role of the funders for the present study. | 13 |

*Items relevant only to the development of a prediction model are denoted by D, items relating solely to a validation of a prediction model are denoted by V, and items relating to both are denoted D;V. We recommend using the TRIPOD Checklist in conjunction with the TRIPOD Explanation and Elaboration document
